# Soil and Water Warming Accelerates Phenology and Down-Regulation of Leaf Photosynthesis of Rice Plants Grown Under Free-Air CO_2_ Enrichment (FACE)

**DOI:** 10.1093/pcp/pcu005

**Published:** 2014-01-30

**Authors:** Minaco Adachi, Toshihiro Hasegawa, Hiroshi Fukayama, Takeshi Tokida, Hidemitsu Sakai, Toshinori Matsunami, Hirofumi Nakamura, Ryoji Sameshima, Masumi Okada

**Affiliations:** ^1^Center for Global Environmental Research, National Institute for Environmental Studies, Tsukuba, 305-8506 Japan; ^2^Agro-Meteorology Division, National Institute for Agro-Environmental Sciences, Tsukuba, 305-8604 Japan; ^3^Department of Agriculture, Kobe University, Kobe, 657-8501 Japan; ^4^Carbon and Nutrient Cycling Division, National Institute for Agro-Environmental Sciences, 3-1-3 Kannondai, Tsukuba, Ibaraki, 305-8604 Japan; ^5^Akita Prefectural Agricultural Experiment Station, Akita, 010-1231 Japan; ^6^Taiyokeiki Co. Ltd., Kita-ku, Tokyo, 114-0032 Japan; ^7^National Agricultural Research Organization, National Agricultural Research Center for Tohoku Region, Morioka, 020-0198 Japan; ^8^Faculty of Agriculture, Iwate University, Morioka, 020-8550 Japan; ^9^Present address: Graduate School of Agriculture, Hokkaido University, Sapporo, 060-8589 Japan.

**Keywords:** Ecosystem warming, FACE (free-air CO_2_ enrichment), *Oryza sativa*, Photosynthetic down-regulation

## Abstract

To enable prediction of future rice production in a changing climate, we need to understand the interactive effects of temperature and elevated [CO_2_] (E[CO_2_]). We therefore examined if the effect of E[CO_2_] on the light-saturated leaf photosynthetic rate (*A*_sat_) was affected by soil and water temperature (NT, normal; ET, elevated) under open-field conditions at the rice free-air CO_2_ enrichment (FACE) facility in Shizukuishi, Japan, in 2007 and 2008. Season-long E[CO_2_] (+200 µmol mol^−1^) increased *A*_sat_ by 26%, when averaged over two years, temperature regimes and growth stages. The effect of ET (+2°C) on *A*_sat_ was not significant at active tillering and heading, but became negative and significant at mid-grain filling; *A*_sat_ in E[CO_2_]–ET was higher than in ambient [CO_2_] (A[CO_2_])–NT by only 4%. Photosynthetic down-regulation at E[CO_2_] also became apparent at mid-grain filling; *A*_sat_ compared at the same [CO_2_] in the leaf cuvette was significantly lower in plants grown in E[CO_2_] than in those grown in A[CO_2_]. The additive effects of E[CO_2_] and ET decreased *A*_sat_ by 23% compared with that of A[CO_2_]–NT plants. Although total crop nitrogen (N) uptake was increased by ET, N allocation to the leaves and to Rubisco was reduced under ET and E[CO_2_] at mid-grain filling, which resulted in a significant decrease (32%) in the maximum rate of ribulose-1,5-bisphosphate carboxylation on a leaf area basis. Because the change in N allocation was associated with the accelerated phenology in E[CO_2_]–ET plants, we conclude that soil and water warming accelerates photosynthetic down-regulation at E[CO_2_].

## Introduction

Human activities have increased the concentration of atmospheric carbon dioxide ([CO_2_]) from the pre-industrial level of 280 µmol mol^−1^. Global annual mean [CO_2_] is now approaching the milestone level of 400 µmol mol^−1^ and is projected to increase to 470–570 µmol mol^−1^ by the middle of the century, despite various mitigation measures being implemented (Fisher et al. 2007). Not only does rising [CO_2_] drive changes in the global environment (e.g. in temperature and precipitation), it also promotes leaf-level photosynthesis and thereby can increase biomass production and grain yield of crops (Long et al. 2004). This CO_2_ fertilization effect is one of the few expected positive impacts of climate change on crop production (Parry et al. 2005), including that of rice, the most important global food crop that feeds more than half of the world’s population. Temperature changes also affect plant growth, but the effects on growth and yield can be positive or negative depending on the current temperature levels and the magnitude of the temperature change (Easterling et al. 2007). We need to better understand the interactive effects of elevated [CO_2_] and temperature to be able to predict future crop production (Lobell and Gourdji 2012).

The central process affected by the interaction between elevated [CO_2_] and temperature is photosynthesis. Theoretical analysis suggested that photosynthetic enhancement due to elevated [CO_2_] may become more pronounced as temperature increases (Long 1991); however, under actual field conditions this positive interaction may not be observed because of other direct and indirect temperature effects. A recent analysis of free-air CO_2_ enrichment (FACE) studies in rice indicated that higher growth temperatures resulted in a smaller yield response to elevated [CO_2_] (Hasegawa et al. 2013). This strongly suggests the need for field trials to investigate the effects of both [CO_2_] and temperature.

Acclimation of photosynthesis to elevated [CO_2_], in which plants grown in CO_2_ enrichment have a different photosynthetic response to elevated [CO_2_] from that of plants grown in ambient air, has been reported in many C_3_ plant species (Ainsworth and Rogers 2007). Reduced photosynthetic enhancement, or down-regulation, is common across many C_3_ crops, including rice, and has been confirmed under open-field conditions (Seneweera et al. 2002, Chen et al. 2005). The scale of down-regulation at elevated [CO_2_] may vary with environmental factors, but only limited information is available on the effects of environmental factors with a potential influence such as temperature or water (Hatfield et al. 2011). In a study using open-top chambers, Lin et al. (1997) showed that rice grown under elevated temperature (ambient + 4°C) showed a reduced stimulation of photosynthesis to elevated [CO_2_] after flowering compared with that grown in ambient temperature, which was probably due to limited sink strength as affected by higher floret sterility under a higher temperature and [CO_2_] condition. The effect of temperature on the scale of down-regulation, however, has not been examined in open fields.

For irrigated rice, which accounts for about 75% of the world’s rice production (Maclean et al. 2002), water temperature is the major factor that controls the thermal environment. Phenology and morphology are directly influenced by water temperature more than by air temperature (Shimono et al. 2007). Advanced phenology by warmer soil and water may accelerate leaf senescence and/or metabolite translocation from the leaves to the grains, because the leaves are the major source of the grain nitrogen (N) (Mae 1997). On the other hand, warmer soil and water will enhance soil N mineralization, which may potentially improve plant N nutrition. Leaf N content is often reduced by elevated [CO_2_] (Long et al. 2004), which is one of the key factors for photosynthetic down-regulation at elevated [CO_2_] in rice (Makino et al. 1997, Sakai et al. 2006), but the combined effects of elevated [CO_2_] and warming on leaf photosynthesis are difficult to predict because of the multiple effects of warming on phenology, soil N supply, plant N uptake and partitioning. These effects need to be tested under field conditions.

In the present study, we therefore investigated the effects of elevated [CO_2_] (200 µmol mol^−1^ above the ambient level) and increased soil and water temperatures (by 2°C) on the light-saturated leaf photosynthetic rate (*A*_sat_) under open-field conditions in the rice FACE facility at Shizukuishi (Japan) for two growing seasons, with the soil and water temperature treatments nested within the [CO_2_] treatment. Our aim was to understand the combined effects of these two factors on leaf photosynthesis via changes in leaf N status at three important crop growth stages during the life cycle of the rice plants: active tillering, heading and mid-grain filling.

## Results

**Fig. 1:**
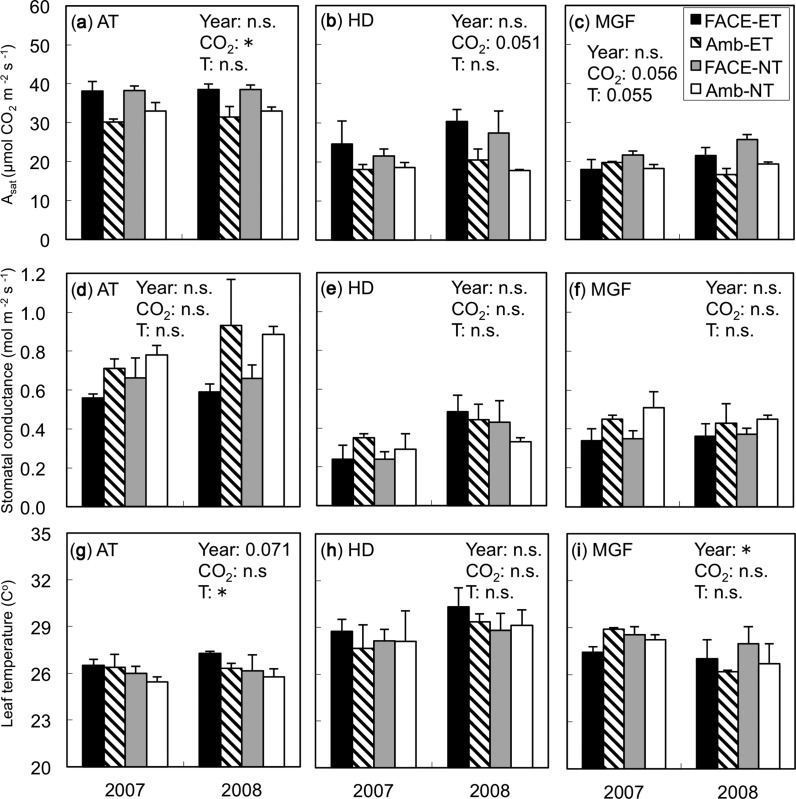
Light-saturated photosynthetic rate (*A*_sat_) (a, b and c), stomatal conductance (d, e, and f) and leaf temperature (g, h and i) in rice grown in 2007 and 2008 at either elevated [CO_2_] (FACE) or ambient [CO_2_] (Amb) and at elevated (ET) or normal (NT) soil and water temperatures. All three parameters were measured at the same [CO_2_] at which plants were grown. Analysis was performed at three growth stages: active tillering (AT; a, d and g), heading (HD; b, e and h) and mid-grain filling (MGF; c, f and j). For significant results, ANOVA *P*-values are indicated in each panel (*P < 0.05, n.s., not significant): year as the main plot, [CO_2_] as the split plot, and soil and water temperatures (T) as the split–split plot. Values are the means ± SE (*n* = 3). No interaction effects were significant.

In both years, the light-saturated leaf photosynthetic rate (*A*_sat_) measured at each growth [CO_2_] condition decreased as the crop growth stage advanced (Fig. 1a–c). Elevated [CO_2_] (E[CO_2_]) increased *A*_sat_ at all growth stages when compared at each soil and water temperature (*P* < 0.05 at active tillering, *P* = 0.051 at heading and *P* = 0.056 at mid-grain filling), with an average increase of 26%. The effect of soil and water warming on *A*_sat_ was not significant at active tillering or heading (Fig. 1a, b). The *A*_sat_ increase due to E[CO_2_] was slightly higher at elevated soil and water temperatures (ET) than at normal temperatures (NT) (25% vs. 16%, respectively, at active tillering, and 42% vs. 35% at heading), but the interaction between [CO_2_] and temperature was not significant. At mid-grain filling, however, the effect of ET on *A*_sat_ became negative (Fig. 1c; *P* = 0.055). The *A*_sat_ increase due to E[CO_2_] averaged over two years became smaller (particularly at ET) at mid-grain filling: 4% with ET and 24% with NT (Fig. 1c). The combined effects of E[CO_2_] and ET on *A*_sat_ averaged about 4% higher than ambient [CO_2_] (A[CO_2_]) and NT, although there was no interaction between [CO_2_] and temperature.

For both years, the effects of E[CO_2_] and ET on stomatal conductance were not significant at any of the three growth stages (Fig. 1d–f), but, for five of six occasions, there was a tendency for stomatal conductance to be lower under E[CO_2_] compared with A[CO_2_]. Leaf temperatures during the gas exchange measurements were generally higher at heading and mid-grain filling compared with those at active tillering, but did not differ between the [CO_2_] and temperature treatments except at active tillering, where that in ET was higher than in NT by 0.8°C (Fig. 1g–i).

**Fig. 2:**
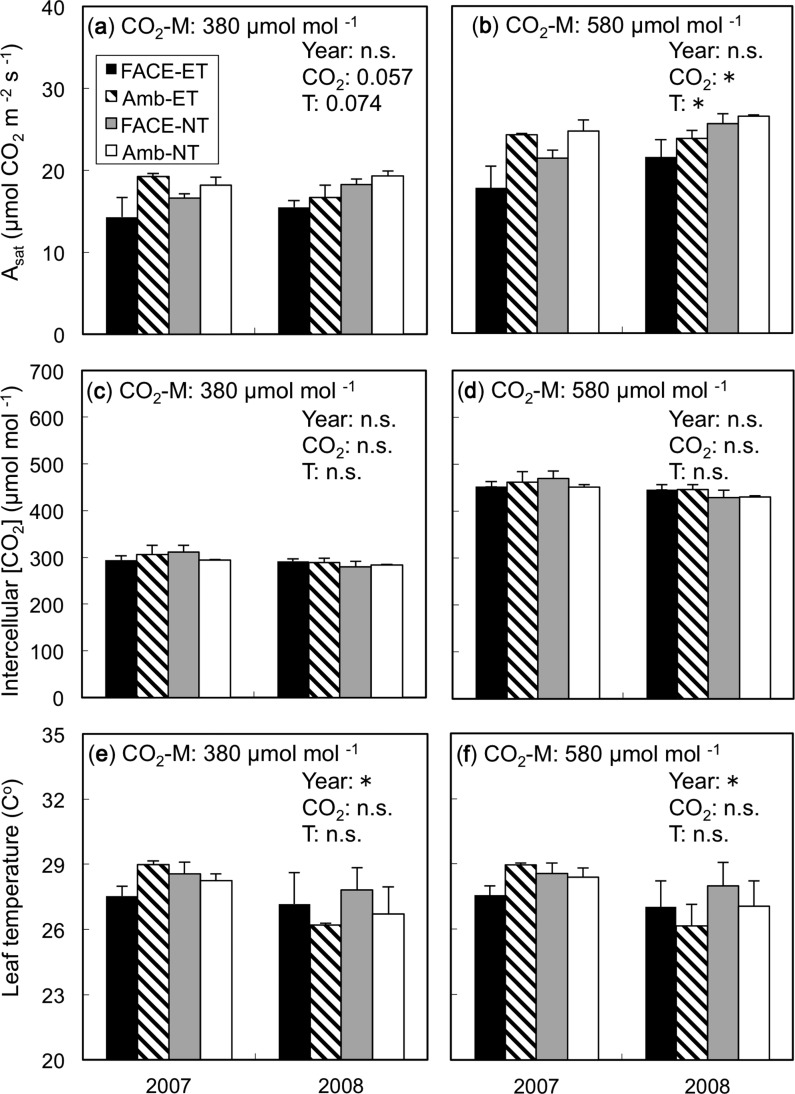
Short-term responses of *A*_sat_, intercellular [CO_2_] and leaf temperature measured under a CO_2_ concentration ([CO_2_]-M) of (a) 380 and (b) 580 µmol mol^−1^ at mid-grain filling. Plants were grown at elevated (FACE) or ambient (Amb) [CO_2_] and elevated (ET) or normal (NT) soil and water temperatures. *A*_sat_, intercellular [CO_2_] and leaf temperature were measured at the same CO_2_ concentrations ([CO_2_]-M) regardless of CO_2_ concentrations during plant growth. For significant results, ANOVA *P*-values are indicated in each panel (*P < 0.05, n.s., not significant): year as the main plot, growth [CO_2_] as the split plot, and soil and water temperatures (T) as the split–split plot. Values are the means ± SE (*n* = 3). No interaction effects were significant.

When *A*_sat_ was compared at the same [CO_2_] in the leaf cuvette ([CO_2_]-M), it was not different between rice grown at E[CO_2_] and A[CO_2_] at active tillering or heading, suggesting that photosynthetic down-regulation was not apparent at these growth stages (data not shown). At mid-grain filling, however, *A*_sat_ of rice grown in E[CO_2_] was significantly lower than *A*_sat_ of rice grown at A[CO_2_] at both [CO_2_]-M levels (Fig. 2a, 380 µmol mol^−1^, *P* = 0.057; Fig. 2b, 580 µmol mol^−1^, *P* < 0.05). The effect of ET on *A*_sat_ was negative at [CO_2_]-M (at 380 µmol mol^−1^, *P* = 0.074; 580 µmol mol^−1^, *P* < 0.05; Fig. 2). There was no significant interaction between [CO_2_] and temperature, but the additive effects of [CO_2_] and temperature decreased the *A*_sat_ of E[CO_2_]–ET plants by as much as 28% in 2007 and 19% in 2008 compared with that of A[CO_2_]–NT plants (Fig. 2b). Growth [CO_2_] conditions did not affect intercellular [CO_2_] or leaf temperature at each [CO_2_]-M (Fig. 2c–f), even where *A*_sat_ was different (Fig. 2a, b), suggesting that down-regulation of *A*_sat_ was not associated with the changes in stomatal conductance.

**Fig. 3:**
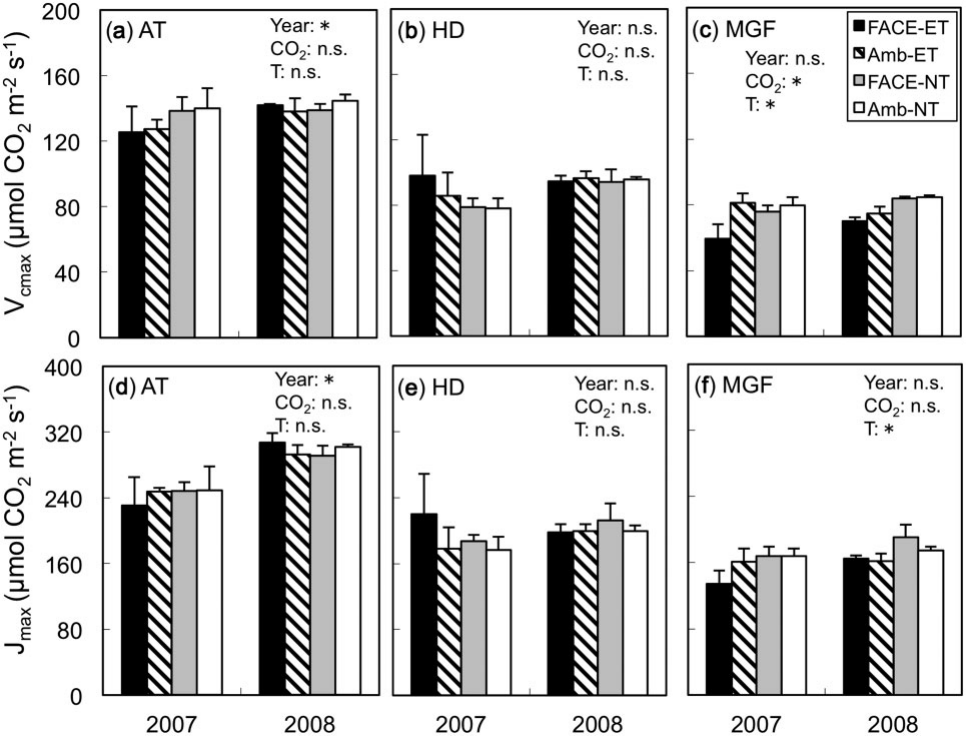
The maximum rates of ribulose-1,5-bisphosphate (RuBP) carboxylation (*V*_cmax_) and electron transport driving RuBP regeneration (*J*_max_) in 2007 and 2008 in the four treatment plots: elevated (FACE) or ambient (Amb) [CO_2_] and elevated (ET) or normal (NT) soil and water temperatures at three growth stages (AT, active tillerling; HD, heading; and MGF, mid-grain filling). For significant results, ANOVA *P*-values are indicated in each panel (*P < 0.05, n.s., not significant): year as the main plot, [CO_2_] as the split plot, and soil and water temperatures (T) as the split–split plot. Values are the means ± SE (*n* = 3).

The maximum rate of ribulose-1,5-bisphosphate carboxylation (*V*_cmax_) and the light-saturated potential rate of electron transport (*J*_max_) did not differ between the CO_2_ and temperature treatments at active tillering or heading (Fig. 3a, b, d, e). At mid-grain filling, E[CO_2_] significantly decreased *V*_cmax_ compared with A[CO_2_] (Fig. 3c; *P* < 0.05), whereas the effect of [CO_2_] was not significant for *J*_max_ (Fig. 3f). At the same stage, ET significantly decreased both *V*_cmax_ (19%) and *J*_max_ (17%) compared with those at NT (*P* < 0.05; Fig. 3c, f). The effects of ET tended to be more pronounced in E[CO_2_], but the interactions between [CO_2_] and temperature were not statistically significant.

**Fig. 4:**
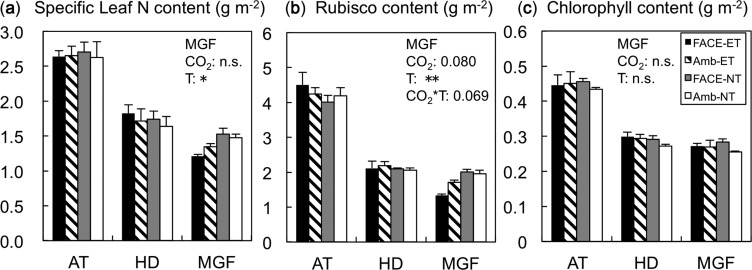
Specific leaf N (a), Rubisco (b) and Chl (c) contents on a leaf area basis in the four treatment plots at three growth stages (AT, active tillerling; HD, heading; and MGF, mid-grain filling) in 2008. The 2008 data are shown because all three measurements were made in 2008 only. In 2007, Rubisco and Chl contents were measured, and the results were similar to those in 2008. For significant results, ANOVA *P*-values at mid-grain filling are indicated in each panel (**P < 0.01; *P < 0.05; n.s., not significant): year as the main plot, [CO_2_] as the split plot, and soil and water temperatures (T) as the split–split plot (*n* = 3). No interaction effects were significant.

Specific leaf N (leaf N content per unit leaf area, SLN), Rubisco and Chl contents on a leaf area basis were not influenced by [CO_2_] and temperature at active tillering and heading (Fig. 4; 2008 data). However, at mid-grain filling, ET significantly decreased SLN content (Fig. 4a; *P* < 0.05) and Rubisco content (Fig. 4b; *P < *0.01) in comparison with NT. At the same stage, E[CO_2_] decreased Rubisco content (*P* = 0.08). The interaction between [CO_2_] and temperature was also significant (Fig. 4b; *P* = 0.069); this resulted in a 32% reduction in Rubisco by E[CO_2_]–ET in comparison with A[CO_2_]–NT. In contrast, Chl content was not affected by either treatment at any of the growth stages (Fig. 4c).

**Fig. 5:**
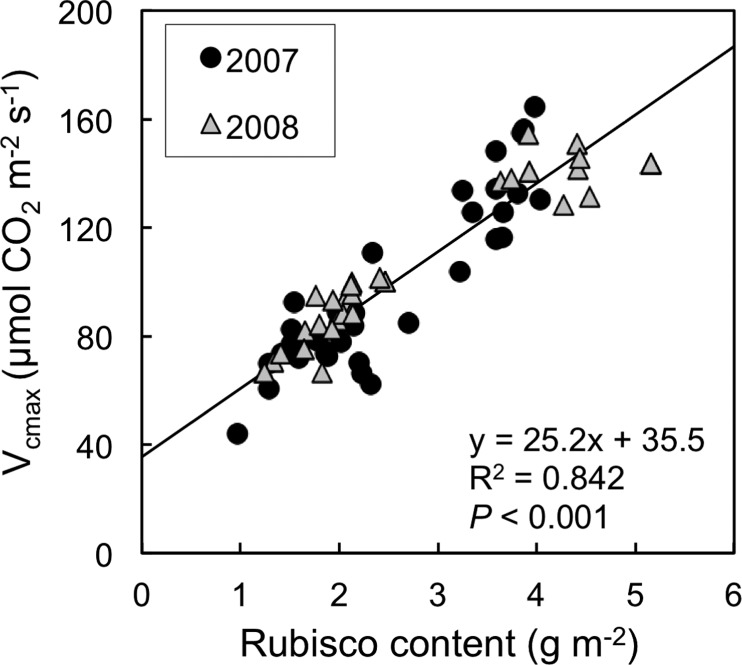
Relationship between Rubisco content and *V*_cmax_. Data for each replicate from two [CO_2_] levels, two temperature levels and three growth stages are shown for each year. A single regression line is drawn for the two years (*n* = 72).

The ratio of *V*_cmax_ to Rubisco content was also significantly decreased by E[CO_2_]–ET as evidenced by a significant interaction between [CO_2_] and temperature at mid-grain filling (*P* < 0.05 in 2007, *P* < 0.001 in 2008; data not shown). Nevertheless, Rubisco content showed a positive correlation with *V*_cmax_ (Fig. 5; *P* < 0.001), consistent in the data sets for the two years. The residuals of the regression line did not suggest any systematic error associated with the treatments (data not shown).

**Fig. 6:**
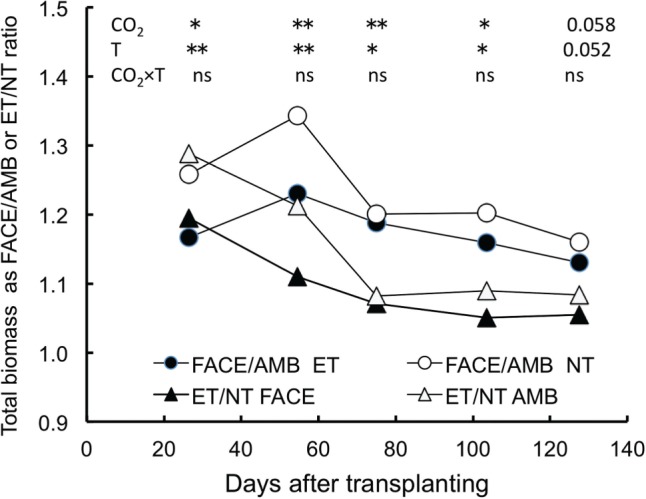
Changes in total crop biomass response to elevated [CO_2_] (FACE/AMB) or ET (ET/NT) averaged over two seasons. Five time points correspond to: 1, early tillering (mid-June); 2, panicle initiation (mid-July); 3, heading (early August); 4, mid-grain filling (early September); and 5, maturity (late September). For significant results, ANOVA *P*-values are indicated at each time point (**P < 0.01; *P < 0.05; n.s., not significant).

**Fig. 7:**
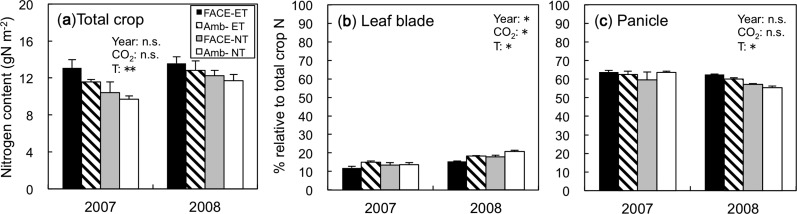
Total crop N content in above-ground parts and roots per land area at mid-grain filling (a), leaf blade N as a percentage of the total crop N content (b) and panicle N as a percentage of the total crop N content at mid-grain filling in 2007 and 2008. ANOVA *P*-values are indicated in each panel (**P < 0.01; *P < 0.05; n.s., not significant): year as the main plot, [CO_2_] as the split plot, and soil and water temperatures (T) as the split–split plot. Values are the means ± SE (*n* = 3).

Both E[CO_2_] and ET had significant and positive effects on total biomass at all growth stages without significant interactions, but the effects were noted in the early stages and decreased as the crop aged; E[CO_2_] increased total biomass in ET and NT by 23% and 34% around panicle initiation (55 days after transplanting, DAT), by 19% and 20% at heading (75 DAT) and by 13% and 16% at maturity (128 DAT), respectively (Fig. 6). Total crop N content (on a land area basis) was increased an average of between 14% and 16% by ET at all growth stages in both years (data at mid-grain filling are shown in Fig. 7). On the other hand, E[CO_2_] only increased total crop N content, except at panicle initiation (*P* = 0.058), when we observed a positive interaction between elevated [CO_2_] and ET (*P* < 0.05, data not shown). Nitrogen in the leaf blades as a percentage of that in the total crop N ranged between 54% and 62% at active tillering and decreased to between 39% and 48% at heading (data not shown). At mid-grain filling, this percentage further decreased to 12–21% as a result of N translocation to the grains (Fig. 7b, c), and was lowest in E[CO_2_]–ET.

## Discussion

We showed for the first time that the down-regulation of photosynthesis in rice due to elevated [CO_2_] was accelerated by soil and water warming under open-field conditions. This suggests that projected increases in temperature may reduce the expected photosynthetic enhancement under elevated [CO_2_] in the late stages of growth. The reduced enhancement of photosynthesis by E[CO_2_] with ET at mid-grain filling was not due to stomatal limitation, because the soil and water warming treatment did not affect stomatal conductance (Fig. 1) or intercellular [CO_2_] (Fig. 2). Recently, a soybean FACE experiment combined with canopy warming by arrays of infrared heaters showed that warming by 3.5°C reduced the stimulatory effect of elevated [CO_2_] on photosynthesis (Ruiz-Vera et al. 2013), but this was associated with reductions in stomatal conductance and intercellular [CO_2_] concentration. Direct comparison between their study and ours is not possible because of the differences in the warming treatment (canopy only vs. soil and water only), ecosystems (dry vs. wet) and crop species (soybean vs. rice), though stomatal limitation could be pronounced where the canopy is directly heated and/or crops are grown under non-flooded conditions as seen in the soybean FACE study of Ruiz-Vera et al. (2013). In contrast, the down-regulation of photosynthesis observed in ET in this study was biochemical and largely associated with a reduction of *V*_cmax_ but not with *J*_max_ (Fig. 3). In the soybean FACE study, Rosenthal et al. (2013) showed a large reduction in *J*_max_ but not *V*_cmax_. Again, the reasons for these differences are difficult to determine without further experiments, but these studies, being the only experiments that examined the combined effects of elevated [CO_2_] and temperature in the open field, suggest that a combination of both warmer canopy and soil and water may cause an even larger reduction in photosynthetic enhancement due to elevated [CO_2_]; this needs to be tested in the future.

Reduction of *V*_cmax_ due to E[CO_2_] and ET at the mid-grain filling stage was largely associated with the reduction in Rubisco content (Fig. 4); a 32% reduction in E[CO_2_]–ET in comparison with A[CO_2_]–NT. SLN content also showed a similar reduction, suggesting that ET and E[CO_2_] altered the crop N budget and thereby influenced the leaf biochemical properties. We predicted that the ET treatment enhanced soil carbon and N metabolism and increased N supply to the plants. In fact, ET increased soil N mineralization by 17% measured within each plot (Tokida et al. 2010), and total crop N uptake by 16% at mid-grain filling (Fig. 7a). This increase, however, did not translate into a higher leaf N content per unit land area (Fig. 7b).

Phenology can affect leaf N status and photosynthetic capacity via two processes: (i) changes in N translocation in response to the N demand from the grains; and (ii) accelerated leaf senescence. Ample evidence exists that the leaf blades are the major source of N to the grain (reviewed by Ma 1997). In fact, more N was allocated to the grains in the E[CO_2_]–ET plot compared with that in NT as a result of earlier onset of grain filling (Fig. 6c). This in part accounts for the reduced SLN in the E[CO_2_]–ET treatment. The reduction in Rubisco content by E[CO_2_]–ET was more pronounced than the reduction in SLN, yet leaf Chl content was not changed either by E[CO_2_] or by ET. A faster decrease in Rubisco content than in Chl content has been reported under an elevated growth temperature (Nagai and Makino 2009), and in the course of senescence at ambient temperature (Uchida et al. 1982, Kumagai et al. 2009). This suggests that accelerated senescence caused by E[CO_2_] and temperature is associated with the changes in gas exchange and biochemical properties during grain filling. In our field experiments, these two processes probably occurred simultaneously, and it is difficult to determine the relative contributions of the two processes, but they over-rode the advantage of greater N uptake to cause the photosynthetic down-regulation in E[CO_2_]–ET.

The crop-level response to the treatments showed advanced phenology; the heading stage was 4 d earlier at ET than at NT, and 1 d earlier in E[CO_2_] than in A[CO_2_] (Tokida et al. 2010). The reduction in days from transplanting to heading in E[CO_2_]–ET amounted to 7–8% when compared with A[CO_2_]–NT. A common projection of future grain yield under global climate change by crop models is that increases in temperature reduce growth duration, which will decrease biomass production and crop productivity (e.g. Parry et al. 2005). Our study further suggests that the advanced phenology reduces N allocation to the leaves and therefore reduced the N available for Rubisco synthesis, which in turn limits the photosynthetic capacity response to E[CO_2_]; this factor has been overlooked in most crop models.

Ample evidence exists for the N-associated down-regulation of photosynthesis under elevated [CO_2_] in rice (Nakano et al. 1997, Makino and Mae 1999, Sakai et al. 2006). Makino and Mae (1999) reported that reduced enhancement of photosynthesis by elevated [CO_2_] was associated with decreases in SLN and Rubisco contents. These changes are largely accounted for by N allocation at the whole-crop level (Makino et al. 1997). The present study showed that advanced phenology as a result of warming could accelerate this process. Interestingly, plants in the NT treatment did not show strong down-regulation in 2007 and 2008, in contrast to the data for the 1999 growing season at Shizukuishi, where the *A*_sat_ of the rice plants in E[CO_2_] was about 30% lower than that in A[CO_2_] at heading when compared at the same [CO_2_]-M (Seneweera et al. 2002). In our present study and that of Seneweera et al. (2002), the N application regimes differed slightly: in the latter, ammonium sulfate was split-applied three times, whereas we applied controlled-release urea (CRU) only once (prior to planting). Comparison of these N regimes in the same years (2003 and 2004) showed that crop N uptake continued after heading in the CRU plot but not in the ammonium sulfate plot (Shimono et al. 2008), suggesting that N nutrition at the grain-filling stage was improved by CRU. This could be the reason for the limited acclimation in the NT plot in the present study. Possibilities for improvement of the crop responses to elevated [CO_2_] under warmer climates via N management practices will be an important subject for future studies.

The whole-crop responses to temperature for biomass and grain production are often more complicated than just considering phenology and gas exchange because multiple processes are involved and their responses are usually non-linear in nature with different temperature optima. Some positive effects of warming can be expected in areas where low temperatures limit plant growth, such as in Shizukuishi (growing season average temperature 20°C); a 2°C warming of soil and water enhanced tillering, crop N uptake and leaf area development in the early growth stages, resulting in a slight but significant increase in biomass accumulation (Tokida et al. 2010, this study). The effect of ET on biomass reduced progressively as crop development proceeded. The effect of E[CO_2_] on grain yield persisted both in ET (14%) and in NT (20%) (T. Tokida et al. unpublished data). However, when rice crops are grown at higher temperatures, negative interactions between temperature and [CO_2_] have been reported. For example, grain setting and biomass allocation to the grain under high day or night temperatures are negatively affected under elevated [CO_2_] (Lin et al. 1997, Matsui et al. 1997, Cheng et al. 2010, Madan et al. 2012). Integration of the experimental results (including the data from this study) into improved crop models should improve our ability to predict future crop production under changing climate.

### Conclusions

Our 2 year studiy using the FACE and warming facility showed that total crop N content was increased under elevated temperature as was expected, but that N allocation to the leaves and to Rubisco was reduced by elevated temperature and [CO_2_]. This resulted in a strong down-regulation of *A*_sat_ in comparison with *A*_sat_ at ambient CO_2_ and normal temperature at mid-grain filling. The changes in N allocation may result from changes in phenology and/or senescence accelerated by warmer soil and water. Our results suggest the need for improvement in the integrated and quantitative understanding of the ecosystem-based response to elevated [CO_2_] and temperature.

## Materials and Methods

### Study site and weather conditions

We conducted the FACE experiments at Shizukuishi town, Iwate prefecture located in the northern part of Japan (39°38′, 140°57′E, 210 m above sea level) in 2007 and 2008. The site belongs to a humid continental climate zone with an average annual temperature of 9.4°C and annual precipitation of 1,545 mm. The soil is an Andosol, typical of volcanic areas. During the growing season (end of May–end of September), mean air temperature was 20.0°C in 2007 and 19.3°C in 2008 (near normal for the region). In-season variations in temperature and solar radiation are summarized in Supplementary Table S1. July in 2008 was cooler than in 2007 and minimum temperatures became lower than 15°C for a few days, but none of the plots suffered from chilling damage on spikelet fertility; the percentage of sterile spikelets averaged <5% for both years (T. Tokida et al. unpublished data). The maximum temperature was recorded in early August in both seasons, which coincided with flowering, but never reached a threshold temperature of 35°C for heat-induced spikelet sterility (Kim et al. 1996). Mean daily solar radiation was 15.0 MJ m^−2^ in 2007 and 15.4 MJ m^−2^ in 2008.

### [CO_2_] and temperature treatments

Detailed descriptions of the relevant methods are provided in Okada et al. (2001) and Tokida et al. (2010). Briefly, elevated [CO_2_] treatments were performed in octagonal plots (‘rings’ hereafter) in farmers’ fields. Each ring was 120 m^2^ (12 m in circle diameter). Pure CO_2_ was supplied from emission tubes installed horizontally at about 30 cm above the canopy on the edges of the FACE rings. We monitored [CO_2_], wind direction and wind speed at the center of each ring. CO_2_ was released from the windward sides during the daylight hours (from sunrise to sunset); the target [CO_2_] was 200 µmol mol^−1^ above the ambient [CO_2_]. Rings with ambient [CO_2_] (without CO_2_ fumigation) were used as the control plots. The daytime [CO_2_] averaged over the season was 570 (2007) and 576 µmol mol^−1^ (2008) in the FACE plots and 379 (2007) and 376 µmol mol^−1^ (2008) in the ambient plots (Tokida et al. 2010, Hasegawa et al. 2013). Six rectangular fields (100 m × 30 m each) were grouped into three blocks. In each block, one field was randomly assigned to FACE and the other to the control. The FACE and ambient rings were at least 90 m apart to avoid contamination with CO_2_.

Within each ring, we established a plot with elevated soil and water temperatures in an area of 5.5 m × 2.7 m encircled with corrugated PVC boards. In the treatment area, water-proof silicone heating wires (type CRX, Tokyo Technological Labo Co., Ltd.) was installed on the submerged soil surface between the rows. We used on–off control of the heaters to elevate soil and water temperatures by 2°C (ET) compared with the normal temperature (NT) plots. Warming was terminated about 2 weeks before harvest (September 25, 2007 and September 29, 2008), when the surface water was drained for harvesting. Seasonal mean water temperature was 23.2°C (NT) and 25.1°C (ET) in 2007, and 22.8°C (NT) and 25.4°C (ET) in 2008 (Tokida et al. 2010). We did not measure air temperatures at different heights in the canopy in this experiment, but the effects of the treatment on air temperatures have previously been examined using similar heating systems in the paddy field. We detected about a 1.0°C increase in air temperature at 20 cm, and about a 0.38°C increase in temperature at 35 cm above the water surface in the warming treatment compared with that in the control plot, only when wind speed is low (<0.7 m s^−1^) and air turbulence was limited; this occurred mostly at night. At the height of flag leaves, there was no difference between the treatments (M. Fukuoka, M. Yoshimoto and T. Hasegawa unpublished data), which was also confirmed by the heat balance model (Yoshimoto 2004). The direct effect of the water warming treatments on the top leaf photosynthesis, therefore, was considered to be negligible. The treatments were laid out in a split-plot design with three replicates, with [CO_2_] as the main plots and the soil and water temperatures as the subplots.

### Crop management

Pre-germinated seeds of a *japonica* cultivar ‘Akitakomachi’ were sown on April 23, 2007 and on April 24, 2008. We raised seedlings in two different chambers under ambient and elevated [CO_2_] (200 µmol mol^−1^ above ambient), and transplanted to the ambient or FACE plots, respectively, at a spacing of 17.5 cm × 30 cm (19.1 hills m^−2^) on May 23, 2007 and May 22, 2008. We planted three seedlings per hill (i.e. a group of seedlings transplanted to one spot). All plots received equal amounts of fertilizers as basal dressing: 9 g m^−2^ of N (3 g m^−2^ as ammonium sulfate and 6 g m^−2^ as coated urea, type LP-70, Chisso-asahi Fertilizer Co., Ltd.; now JCAM Agri. Co., Ltd.), 12.5 g m^−2^ of K (7.5 g as KCl and 5.0 g as potassium silicate) and 13.1 g m^−2^ of P as fused magnesium phosphate.

### Gas exchange measurements

We conducted the gas exchange measurements on the most recently fully expanded leaves at three growth stages in 2007 and 2008: late June to early July (active tillering, AT), early August (heading, HD) and late August (mid-grain filling, MGF). Before the measurements, we measured the Chl content in 4–6 leaves per treatment plot non-destructively, using a Chl meter (SPAD-502, Konica Minolta Optics, Inc.). We then used one or two leaves with representative Chl content for the gas exchange measurements by using a portable photosynthesis system with blue and red LED light sources (LI-6400, LI-COR Bioscience). All measurements were conducted between 08:00 and 15:00 h on clear days; block temperature in the cuvette was fixed at 25°C, and the photosynthetic photon flux density was fixed at 1,800 µmol m^−2 ^s^−1^, with a flow rate of 500 µmol s^−1^. The 6400-01 CO_2_ injector attached to the main system was used to control [CO_2_] in the cuvette. First, *A*_sat_ at the respective [CO_2_] (380 µmol mol^−1^ for the control plants and 580 µmol mol^−1^ for the FACE plants) was measured. Then, [CO_2_] conditions in the cuvette were switched to the opposite conditions to determine short-term responses of *A*_sat_ to [CO_2_]. [CO_2_] was then changed to 50 µmol mol^−1^. After the stomatal conductance had stabilized, we measured *A*_sat_ at different [CO_2_] of 50, 100, 150, 250, 380, 580, 800, 1,000 and 1,200 µmol mol^−1^ to determine the relationship between *A*_sat_ and intercellular [CO_2_] (*C*_i_). At each growth stage, all measurements across treatments took 3 d. Within each day, measurements for all four treatments were completed in one block to avoid possible confounding effects of day to day variations.

*V*_cmax_ and *J*_max_ were calculated by fitting the equations of Farquhar et al. (1980), following the procedure of Long and Bernacchi (2003). After completion of the gas exchange measurements of the day, we sampled a 3 cm long segment from the middle part of each leaf blade, wrapped it in aluminum foil and froze it in liquid nitrogen. This process was completed within about 10 s for each sample. The samples were stored in a deep freezer at −80°C for analyses of Rubisco and Chl contents. Only in 2008, additional leaf segments were taken from the detached leaves for SLN content determination and oven-dried at 80°C for >72 h.

### Measurements of Chl, Rubisco and specific leaf N contents

The frozen leaf samples were homogenized in a mortar with buffer (3 ml) containing 50 mM HEPES-KOH (pH 7.5), 5 mM MgCl_2_, 5 mM dithiothreitol (DTT), 1 mM EDTA, 4 mM amino-*n*-caproic acid, 0.8 mM benzamidine-HCl, 0.05% (v/v) Triton X-100, 5% glycerol and 0.1% (w/v) polyvinylpolypyrrolidone in the presence of a small amount of quartz sand. A part of the homogenate was mixed with acetone and the solution was adjusted to an acetone concentration of 80% (v/v). This was then used for Chl determination as described by Porra et al. (1989). The remaining homogenate was centrifuged at 15,000×*g* for 2 min, and the supernatant was used for determination of Rubisco content from stoichiometric binding of [^14^C]carboxy-d-arabinitol-1,5-bisphosphate as described previously (Ishikawa et al. 2009). SLN was determined for the dried leaf samples by the Kjeldhal method.

### Plant sampling and crop N determination

Methods for plant samplings and biomass determination at different growth stages were described in Tokida et al (2010); briefly plants from eight hills (equivalent to 0.42 m^2^) were collected from each plot at five times points: early tillering (mid-June), panicle initiation (mid-July), heading (early August), mid-grain filling (early September) and maturity (late September). They were separated into different organs, oven-dried at 80°C, and weighed for biomass determination. The dried samples of different organs were ground and subject to Kjeldahl analysis for N determination.

### Statistical analysis

An analysis of variance (ANOVA) was conducted at each growth stage by applying a split–split plot design, where year was treated as the main factor, [CO_2_] as the split factor and temperature as the split–split factor. We used the MIXED procedure of SAS v 9.2 (SAS Institute Inc.) for the computation. For statistical significance, we used the levels of 0.001, 0.01, 0.05 and 0.1, but in the case of 0.05 < *P* < and 0.1, we present the actual *P*-values.

## Supplementary data

Supplementary data are available at PCP online.

## Funding

This work was supported by the Ministry of Agriculture, Forestry and Fisheries, Japan [through a research project entitled ‘Development of technologies for mitigation and adaptation to climate change in agriculture, forestry and fisheries’]; the Ministry of Education, Culture, Sports, Science & Technology, Japan [through a Grant-in-Aid for Scientific Research on Innovative Areas (No. 22114515)].

## Supplementary Material

Supplementary Data

## Abbreviations

*A*_sat_: light-saturated photosynthetic rate
[CO_2_]: atmospheric CO_2_ concentrations
CRU: controlled-release urea
DAT: days after transplanting
ET: elevated soil and water temperature
FACE: free-air CO_2_ enrichment
*J*_max_: maximum rate of electron transport
NT: normal (ambient) temperature
SLN: specific leaf nitrogen
*V*_cmax_: maximum rate of ribulose-1,5-bisphosphate carboxylation
