# Supplementary material for: Soil and Water Warming Accelerates Phenology and Down-Regulation of Leaf Photosynthesis of Rice Plants Grown Under Free-Air CO2 Enrichment (FACE)
Source: Plant Cell Physiol. 2014 Jan 30;55(2):370–80. doi: 10.1093/pcp/pcu005 (PMC3913447; doi:10.1093/pcp/pcu005)
Supplement: Supplementary Data [file supp_55_2_370__index.html]

Soil and water warming accelerates phenology and down-regulation of leaf photosynthesis of the rice plants grown under free-air CO2 enrichment (FACE) — Soil and water warming accelerates phenology and down-regulation of leaf photosynthesis of rice plants grown under free-air CO2 enrichment — Soil and Water Warming Accelerates Phenology and Down-Regulation of Leaf Photosynthesis of Rice Plants Grown Under Free-Air CO2 Enrichment (FACE) — Supplementary Data 

# Soil and Water Warming Accelerates Phenology and Down-Regulation of Leaf Photosynthesis of Rice Plants Grown Under Free-Air CO2 Enrichment (FACE)

## Supplementary Data

files

**Files in this Data Supplement:**

- Supplementary Data - xlsx file
